# Plantar pressure in relation to hindfoot varus in people with unilateral upper motor neuron syndrome

**DOI:** 10.1002/jfa2.12041

**Published:** 2024-09-02

**Authors:** Bente E. Bloks, Lise M. Wilders, Jan Willem K. Louwerens, Alexander C. Geurts, Jorik Nonnekes, Noël L. W. Keijsers

**Affiliations:** ^1^ Department of Research Sint Maartenskliniek Nijmegen The Netherlands; ^2^ Department of Rehabilitation Radboud University Medical Center Donders Institute for Brain, Cognition and Behaviour Nijmegen The Netherlands; ^3^ Department of Rehabilitation Sint Maartenskliniek Nijmegen The Netherlands; ^4^ Department of Orthopedics Sint Maartenskliniek Nijmegen The Netherlands; ^5^ Department of Sensorimotor Neuroscience Donders Institute for Brain Cognition and Behaviour Radboud University Nijmegen The Netherlands

**Keywords:** foot deformity, hindfoot varus, plantar pressure, unilateral upper motor neuron syndromes

## Abstract

**Introduction:**

Hindfoot varus deformity is common in people with unilateral upper motor neuron syndrome (UMNS) and can be dynamic or persistent. The aims of this study were (1) to gain insight into plantar pressure characteristics of people with chronic UMNS in relation to hindfoot varus and (2) to propose a quantitative outcome measure, based on plantar pressure, for the scientific evaluation of surgical interventions.

**Methods:**

In this retrospective study, a cohort comprising plantar pressure data of 49 people with UMNS (22 “no hindfoot varus”, 18 “dynamic hindfoot varus”, and 9 “persistent hindfoot varus”), and 586 healthy controls was analyzed. As an indication of plantigrade foot contact, the ratio between the plantar contact area of the affected and the non‐affected foot was calculated. To investigate spatial and temporal aspects of plantar pressure, normalized plantar pressure patterns and center of pressure trajectories were computed.

**Results:**

People with UMNS had lower plantar pressure area ratios compared to healthy controls. Additionally, increased plantar pressure underneath the lateral foot was found in people with a persistent hindfoot varus. Center of pressure trajectories were more lateral during the first 26% of the stance phase in people with a dynamic hindfoot varus and during the first 82% of the stance phase in people with a persistent hindfoot varus compared to healthy controls.

**Conclusion:**

Spatial and temporal differences in plantar pressure were found in people with dynamic or persistent hindfoot varus deformity. We propose to primarily use the medio‐lateral center of pressure trajectory as outcome measure for the scientific evaluation of surgical interventions targeting hindfoot varus.

## 1 INTRODUCTION

Hindfoot varus deformities are frequently observed in people with chronic unilateral upper motor neuron syndrome (UMNS), such as stroke or traumatic brain injury [1, 2, 3]. They result from a disturbed muscle balance around the foot and ankle joint and can be dynamic or persistent [4]. A dynamic hindfoot varus, arising from muscle weakness and/or spasticity, is not continuously present during standing and walking but can severely affect positioning of the foot at initial contact when present during the terminal swing phase and loading response of walking. This is often caused by a relatively strong tibialis anterior compared to the lateral foot elevators [4]. Prolonged spastic overactivity of the lower leg muscles, most commonly the tibialis posterior, may result in muscle contractures [5], causing dynamic foot deformity evolving into persistent deformity. A persistent hindfoot varus severely impairs stability during the entire stance phase [4].

To improve standing and walking capacity, treatment of hindfoot varus in people with UMNS is essential. For appropriate treatment selection and for the scientific evaluation of surgical interventions, an accurate evaluation of the deformity is crucial [6]. Over the last decades, numerous multi‐segment foot models (MSFM) have been developed to quantitatively evaluate foot function using a three‐dimensional instrumented gait analysis. However, only a limited number of validation studies have been performed since a direct comparison with the underlying bone structures is challenging and can only be achieved with a sophisticated videofluoroscopy setup or by in vitro or in vivo bone pin measurements [7]. Studies examining the accuracy of MSFMs have reported moderate results for measuring hindfoot varus/valgus angles, indicated by a root mean squared error of 3.6° compared to videofluoroscopy [8] and an error of up to 4.8° compared to bone mounted markers [9]. Moreover, performing an instrumented gait analysis is very time consuming and requires the availability of a three‐dimensional motion lab.

An alternative method for evaluating foot function is plantar pressure assessment [10, 11, 12, 13, 14, 15]. Research indicates that in people with chronic UMNS, peak pressures underneath the forefoot are lower compared to those in healthy controls [10, 11, 15]. Additionally, a diminished plantar contact area has been observed for the affected foot compared to the non‐affected foot in people with UMNS [13]. However, previous studies included people with chronic UMNS, irrespective of foot deformities, or did not distinguish between different types of foot deformities [10, 11, 12, 13, 14, 15], which makes it difficult to draw conclusions on the clinical value of plantar pressure measurements for the evaluation of foot deformities. Furthermore, prior studies that investigated plantar pressure in people with chronic UMNS primarily focused on the spatial aspect of plantar pressure [10, 11, 12, 13, 14, 15], whereas most studies lacked evaluations of the temporal aspect. Especially for dynamic foot deformity, relying solely on spatial analyses of plantar pressure could potentially lead to underestimation of the deformity, as dynamic deformities may only be present during short periods within the stance phase. Therefore, a temporal investigation of plantar pressure may provide more profound insights here. Hence, a comprehensive evaluation examining the relationship between foot deformities and both spatial and temporal plantar pressure characteristics would be a valuable initial step to investigate the clinical value of plantar pressure measurements for the evaluation of hindfoot varus deformities in people with UMNS.

The aims of this study were (1) to gain insight into spatial and temporal plantar pressure characteristics of people with chronic UMNS in relation to dynamic and persistent hindfoot varus and (2) to propose a quantitative outcome measure based on plantar pressure for the scientific evaluation of surgical interventions. We hypothesized that the difference in plantar contact area between the affected and the non‐affected foot would increase with the severity of hindfoot varus, that is, the greatest difference in persistent hindfoot varus, followed by dynamic hindfoot varus and, ultimately, no hindfoot varus. Additionally, we hypothesized to find increased pressure underneath the lateral forefoot and decreased pressure underneath the central and medial forefoot in the case of dynamic or persistent hindfoot varus, as seen in other neurological disorders with hindfoot varus [16, 17]. Regarding the temporal aspect of plantar pressure, we hypothesized lateralized center of pressure throughout the gait phases when hindfoot varus is present, as has been observed in healthy subjects walking with an inverted foot position [18].

## 2 MATERIALS AND METHODS

### 2.1 Participants and design

In this retrospective study, adults with chronic unilateral UMNS, including stroke and traumatic brain injury, who had visited the mobility outpatient clinic of the Gait Expertise Center of the Sint Maartenskliniek between March 2018 and October 2022 for the assessment of neurological gait impairments, were screened for inclusion. Chronic UMNS was defined as at least 6 months post onset of the brain lesion. Only subjects with at least three plantar pressure measurements of both feet and adequate photographs and video recordings of the affected foot were included. The exclusion criteria were: (1) previous foot/ankle surgery on the affected side, (2) a previous foot/ankle fracture on the affected side, and (3) comorbidities that might cause ankle‐foot deformity. In addition, a group of 586 healthy controls without disabilities that might affect their gait, including the presence of foot deformities, were included as a reference. The healthy control data has been described earlier [19] and was expanded by 174 people.

### 2.2 Examinations

Classification of all affected UMNS feet into foot deformity categories was performed based on photographs and video recordings. First, the hindfoot position during stance was classified as “varus” or “no varus” based on photographs of the posterior hindfoot. This was independently done by two raters who discussed their assessment in case of discrepancy to reach consensus. Subsequently, the hindfoot position during gait was classified as “varus” or “no varus” based on close‐up video recordings of the posterior hindfoot during walking. One rater initially assessed these video recordings, which was subsequently discussed with a second rater. Each rater had extensive experience, with at least 2 years of expertise in assessing hindfoot varus in individuals with UMNS. Finally, all affected feet were classified into three subgroups: (1) no hindfoot varus during standing and walking (“no hindfoot varus”), (2) no hindfoot varus during standing, but a varus position of the hindfoot during the loading response phase of walking (“dynamic hindfoot varus”), and (3) a hindfoot varus during standing and the entire stance phase of walking (“persistent hindfoot varus”).

Plantar pressure measurements were performed with a 0.5 m long Footscan pressure plate (RSscan) placed on top of a force plate (Kistler). The pressure plate and force plate were synchronized with an RSscan 3D‐box and plantar pressure patterns were recorded at 500 Hz. Three to six barefoot walking trials were performed for both feet at preferred walking speed.

### 2.3 Data analyses

As indicator of plantigrade loading, the ratio between the plantar contact area of the affected and the non‐affected foot was calculated. This was done by determining the contour of the mean plantar pressure pattern over the stance phase at 0.5 N/cm^2^. After that, the area within the contour was calculated and averaged across three to six walking trials for each foot. Subsequently, the mean plantar contact area of the affected foot was divided by the sum of the mean plantar contact areas of the affected and non‐affected foot, which will be referred to as the plantar pressure area ratio. For healthy controls, it was randomized whether the left or right foot was used as the numerator.

To be able to compare plantar pressure among subjects, plantar pressure patterns were normalized for foot size and foot progression angle according to a previously described method [20]. The focus of this study was on the plantar pressure distribution rather than absolute pressure values, as absolute values are highly influenced by body weight and walking velocity [19]. Therefore, the pressure of each pixel was divided by the sum of the pressures of all pixels. Average normalized plantar pressure patterns over three to six measurements were calculated for each subject and multiplied by the mean total pressure of all subjects. Furthermore, center of pressure trajectories in the medio‐lateral and anterior‐posterior directions were calculated and normalized to foot length and width based on the pressure area. Foot length was defined as the distance between the most posterior part of the heel and the most anterior part of the toes. Foot width was defined as the distance between the most medial and the most lateral part of the forefoot. Finally, stance time was calculated and used as an indicator of walking velocity [21].

### 2.4 Statistical analyses

To assess the validity of the plantar pressure area ratio as outcome measure, analyses were conducted using the data from healthy controls. First, the mean absolute difference between the plantar contact area of the left and right foot was calculated to quantify differences in plantar contact area in individuals without foot deformities. Additionally, *R*‐squared (*R* ^2^) was calculated to examine the correlation between the plantar contact areas of both feet in healthy controls.

One‐way analyses of variance (ANOVA) and post hoc independent samples *t*‐tests with Bonferroni correction were performed to compare the plantar pressure area ratios and stance times between (sub)groups. A *p*‐value lower than 0.05 was considered statistically significant.

To compare the normalized plantar pressure patterns at pixel level, independent samples *t*‐tests were performed. A *p*‐value lower than 0.0125 was considered statistically significant. This *p*‐value was calculated by dividing the initial *p*‐value of 0.05 by the number of clusters of pixels with a similar difference from the plantar pressure pattern of healthy controls, which was determined to be four. This method to correct the *p*‐value for the number of comparisons has previously been used in the analysis of plantar pressure patterns [22] and was initially derived from neuroscience [23].

Center of pressure trajectories were statistically compared between (sub)groups with statistical parametric mapping [24]. One‐way ANOVA and post hoc independent samples *t*‐tests with Bonferroni correction were used to compare the center of pressure trajectories. Statistical significance was defined as *p* < 0.05.

## 3 RESULTS

One hundred and four adults with UMNS were screened for eligibility. Out of these, 92 subjects had at least three plantar pressure measurements of both feet and good photographs and video recordings of the affected foot. Forty‐three subjects were excluded because of previous surgery on the affected foot/ankle (*n* = 39), a previous foot/ankle fracture on the affected side (*n* = 3), or comorbidity that might cause ankle‐foot deformity (*n* = 1). Ultimately, 49 participants were included. Among them, 22 were classified as having “no hindfoot varus”, 18 as having “dynamic hindfoot varus”, and nine with “persistent hindfoot varus”. Table 1 presents the subject characteristics.

**TABLE 1:** Subject characteristics.

Group	*n* (#people)	Sex (#female/#male)	Age (years, mean ± SD)	Affected side (#left/#right)	Type of UMNS (#stroke/#traumatic brain injury)
Healthy controls	586	294/292	49 ± 13	‐	‐
No hindfoot varus	22	8/14	54 ± 11	15/7	18/4
Dynamic hindfoot varus	18	9/9	51 ± 12	9/9	17/1
Persistent hindfoot varus	9	2/7	49 ± 15	5/4	6/3

Abbreviation: UMNS, upper motor neuron syndrome.

### 3.1 Plantar pressure areas

The mean absolute difference between the plantar contact area of the left and right foot for healthy controls was 5.1 ± 3.9 cm^2^. The R‐squared value between the plantar contact area of the left and right foot for healthy controls was 0.86, which implies that 86% of the variance in a plantar contact area can be explained by the plantar contact area of the contralateral foot.

**FIGURE 1:**
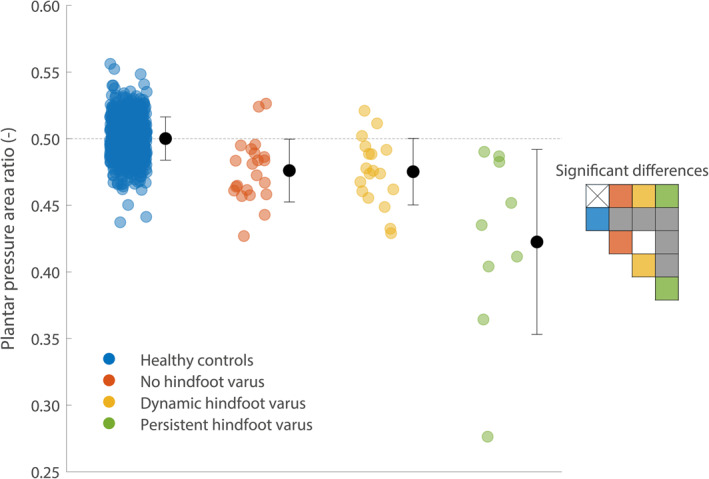
Plantar pressure area ratios. The plantar pressure area ratio is defined as the plantar pressure area of the affected foot divided by the sum of the plantar pressure areas of the affected and non‐affected foot. Plantar pressure area ratios for all feet and the means and standard deviations for each (sub)group are presented. Significant differences between (sub)groups are shown in the table on the right side of the figure. A gray box indicates a significant difference between (sub)groups, whereas a white box indicates no significant difference between (sub)groups.

Figure 1 shows the plantar pressure area ratios. Plantar pressure area ratios were significantly different between (sub)groups (F(3,631) = 72.1, *p* < 0.001). The plantar pressure area ratio was significantly smaller in all UMNS subgroups compared to healthy controls (*p* < 0.001, Figure 1). Furthermore, the plantar pressure area ratio was significantly smaller in the “persistent hindfoot varus” subgroup compared to the “no hindfoot varus” and “dynamic hindfoot varus” subgroups (*p* < 0.001, Figure 1).

### 3.2 Normalized plantar pressure patterns

Seven feet could not be normalized because information about the complete foot shape was lacking due to severe equinus or varus deformity. This included one foot from the “no hindfoot varus” subgroup (plantar pressure area ratio: 0.43), two feet from the “dynamic hindfoot varus” subgroup (plantar pressure area ratios: 0.43, 0.45), and four feet from the “persistent hindfoot varus” subgroup (plantar pressure area ratios: 0.28, 0.36, 0.41, 0.44). Therefore, 21 feet classified as “no hindfoot varus”, 16 feet classified as “dynamic hindfoot varus”, and five feet classified as “persistent hindfoot varus” were included in all subsequent analyses.

**FIGURE 2:**
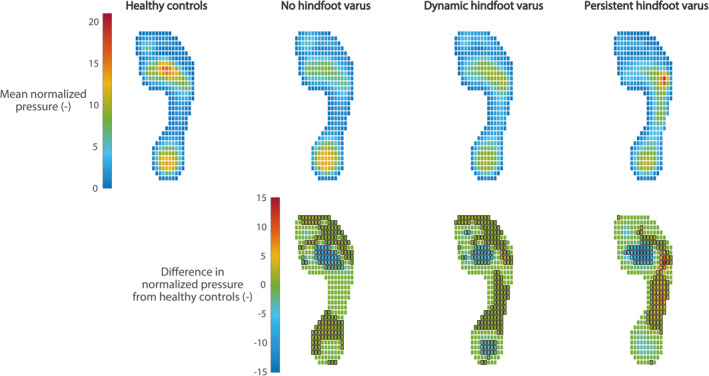
Plantar pressure patterns. The plantar pressure patterns of the unilateral upper motor neuron syndrome subgroups and healthy controls averaged over the stance phase are presented in the top panels. The lower panels present the differences in normalized plantar pressure from healthy controls. Positive values indicate an increase in plantar pressure, whereas negative values indicate a decrease in plantar pressure compared to healthy controls. Pixels with a black border indicate a statistically significant difference (*p* < 0.0125).

Figure 2 presents the normalized plantar pressure patterns. A significant decrease in plantar pressure underneath the second/third metatarsal heads was found for all UMNS subgroups compared to healthy controls. A significant increase in plantar pressure underneath the midfoot was found for the “dynamic hindfoot varus” and “persistent hindfoot varus” subgroups. Furthermore, a significant increase in plantar pressure underneath the fifth metatarsal head was found for the “persistent hindfoot varus” subgroup compared to healthy controls.

### 3.3 Center of pressure trajectories

**FIGURE 3:**
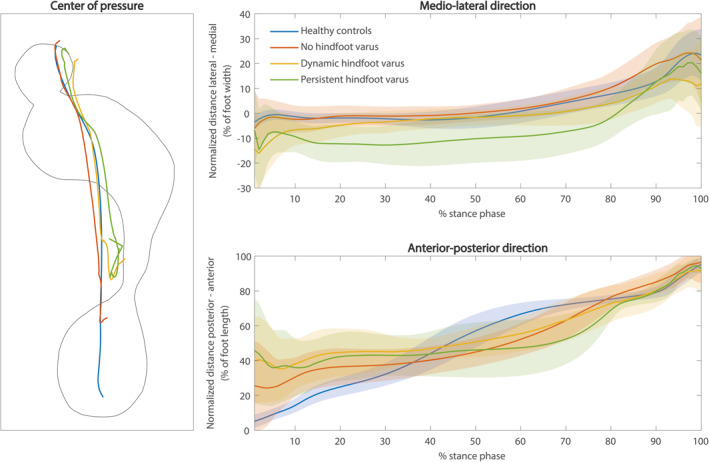
Center of pressure trajectories. In the left panel, the mean center of pressure trajectory is shown for all (sub)groups. In the right panels, the mean center of pressure position ± standard deviation in the medio‐lateral (upper right panel) and anterior–posterior (lower right panel) directions over the stance phase are presented. In the medio‐lateral direction, the *y*‐axis indicates the normalized distance from the medio‐lateral center of the foot, expressed as a percentage of foot width. In the anterior‐posterior direction, the *y*‐axis indicates the normalized distance from the most posterior part of the heel, expressed as a percentage of foot length.

Center of pressure trajectories are shown in Figure 3. In the medio‐lateral direction, the center of pressure trajectory was significantly different between (sub)groups during almost the entire stance phase, except between 92% and 95% of the stance phase (F(3,624) = 4.6, *p* < 0.04). Post hoc tests revealed a lateralized center of pressure during the first 26% of the stance phase for the “dynamic hindfoot varus” subgroup compared to healthy controls (*p* < 0.001). For the “persistent hindfoot varus” subgroup, the center of pressure was significantly more lateral during the first 82% of the stance phase compared to healthy controls (*p* < 0.001).

In the anterior–posterior direction, the center of pressure trajectory was significantly different between (sub)groups during the first 36% and between 44% and 94% of the stance phase (F(3,624) = 4.6, *p* < 0.001). The center of pressures of all UMNS subgroups were more anterior during the first 30% of the stance phase compared to healthy controls (*p* < 0.001). Furthermore, between 53% and 77% of the stance phase, the center of pressures of all UMNS subgroups were more posterior compared to healthy controls (*p* < 0.001). All other statistical parametric mapping results are provided in Supporting Information S1.

Stance time was significantly different between (sub)groups (F(3,624) = 96.1, *p* < 0.001). Stance time was significantly longer for the UMNS subgroups (“no hindfoot varus”: 0.96 ± 0.27 s, “dynamic hindfoot varus”: 0.99 ± 0.35 s, “persistent hindfoot varus”: 0.90 ± 0.09 s) compared to healthy controls (0.67 ± 0.08 s). No significant differences in stance time were found between UMNS subgroups.

## 4 DISCUSSION

This study revealed diminished plantar pressure area ratios among all UMNS subgroups when compared to healthy controls. In the case of persistent hindfoot varus, an increased lateral foot pressure was observed. Notably, the center of pressure was lateralized during the first 26% of the stance phase for a dynamic hindfoot varus and during the first 82% of the stance phase for a persistent hindfoot varus. Additionally, a more anterior foot landing (0%–30% of the stance phase) and a more posterior center of pressure between 53% and 77% of the stance phase was observed in all UMNS subgroups compared to healthy controls. These findings highlight spatial and temporal differences in plantar pressure in relation to hindfoot varus in people with UMNS.

The smaller plantar pressure area ratios in people with UMNS imply reduced plantigrade support on the affected side, which is in line with previous findings [13]. Although a significantly smaller plantar pressure area ratio was found for persistent hindfoot varus compared to all other (sub)groups, the between‐subject variability was high. This variability can be attributed to differences in severity of hindfoot varus between subjects. Additionally, increased midfoot loading caused by hindfoot varus disturbed the plantar pressure area ratio. In some people, substantial plantar pressure was detected underneath the midfoot of their affected foot due to hindfoot varus, whereas no plantar pressure was detected underneath the midfoot of their non‐affected foot. This resulted in only a small difference in plantar contact area between both feet, whereas a large difference in plantar pressure distribution existed. Therefore, the plantar pressure area ratio alone does not seem to be a valuable outcome measure to evaluate hindfoot varus deformity in people with UMNS.

The normalized plantar pressure patterns revealed decreased plantar pressure underneath the central forefoot in all UMNS subgroups, which is consistent with previous findings [10, 15]. Furthermore, spatial deviations in plantar pressure in individuals with a persistent hindfoot varus were found when compared to healthy controls. For people with a dynamic hindfoot varus, the differences from healthy controls were less pronounced. Hence, evaluation of the mean normalized plantar pressure pattern is useful for the assessment of persistent hindfoot varus but seems to be less valuable for the evaluation of dynamic hindfoot varus. Dynamic hindfoot varus manifests only briefly during the stance phase, which means that averaging the plantar pressure pattern over the entire stance phase may consequently result in underestimation of dynamic deformities. To gain further insight into foot function in people with dynamic hindfoot varus, additional analyses focusing on the temporal aspect of plantar pressure should be performed.

The temporal aspect of plantar pressure was evaluated by the center of pressure trajectory. The center of pressure was lateralized when hindfoot varus was present, that is, during the first part of the stance phase for dynamic hindfoot varus and during almost the entire stance phase for persistent hindfoot varus. Furthermore, a more anterior foot strike and a more posterior center of pressure during 53%–77% of the stance phase were observed in all UMNS subgroups compared to healthy controls. The reduced anterior–posterior center of pressure displacement is in agreement with previous findings [25, 26]. Our results indicate that, especially for dynamic foot deformities, investigation of plantar pressure over time provides better insight into the biomechanical deviations compared to investigation of spatial aspects of plantar pressure alone.

A recent systematic review on assessment methods for the evaluation of foot surgery in people following stroke highlighted the need for an accurate and objective method to analyze foot function [6]. Based on our results, we propose to use the medio‐lateral center of pressure trajectory over time as outcome measure for the evaluation of surgical interventions targeting hindfoot varus as it can identify hindfoot varus throughout the stance phase of gait for persistent as well as dynamic deformities. In addition, for the evaluation of persistent hindfoot varus, a comprehensive analysis of the complete plantar pressure pattern could be valuable to gain more insight into the exact location of plantar pressure deviations. Further research is needed to investigate the responsiveness of these outcome measures to surgical interventions. Furthermore, future studies should examine the correlation between these measures and other clinical and functional outcomes to assess their validity as evaluation tools.

A limitation of this study is that not all plantar pressure patterns could be normalized due to a lack of information on the complete foot shape, which was the case for seven people with UMNS in this study with severe equinus or varus deformity. While their exclusion is unlikely to have had a significant impact on the results, as including more severe foot deformities would probably have led to even more pronounced differences between (sub)groups, it underscores a moderate feasibility of the normalization method for plantar pressure patterns in individuals with UMNS. Consequently, future work is needed to develop a normalization method that is applicable to all plantar pressure patterns, including those with severe deviations. Such an approach could involve anthropometric measurements of the foot to overcome the limitation associated with the lack of information on the complete foot shape. Another limitation of this study is that people with UMNS had a longer stance time compared to healthy controls. Since decreased walking velocity is associated with spatial and temporal differences in plantar pressure [21, 27, 28], the results reported in this study could partially be explained by a lower walking speed in people with UMNS as represented by the longer stance time. Nonetheless, stance time was not different between UMNS subgroups, whereas the spatial and temporal plantar pressure characteristics were different between these subgroups. In addition, stance time may not be a valid indicator of walking speed in individuals with UMNS since stance time of the affected leg may be significantly reduced compared to the non‐affected leg [29]. However, stance time was not used to accurately estimate walking speed in this study but only to provide an impression on the potential differences in walking speed between (sub)groups and consequently the influence of these differences on plantar pressure. Lastly, this study related plantar pressure to classification of hindfoot varus deformities, which makes it impossible to study hindfoot varus deformities on a continuous scale. To address this limitation, further research is recommended to validate plantar pressure measurements for studying foot function on a continuous scale, for example by using biplanar videofluoroscopy. This will allow to compare the plantar pressure patterns with the hindfoot angle during gait and can, therefore, provide further insight into the value of plantar pressure measurements for the evaluation of foot deformities.

## 5 CONCLUSION

This study revealed spatial and temporal differences in plantar pressure among individuals with UMNS, in relation to dynamic and persistent hindfoot varus deformity. We propose to use the medio‐lateral center of pressure trajectory as key outcome measure for the scientific evaluation of surgical interventions that aim to correct the hindfoot position. In the case of a persistent hindfoot varus, a comprehensive analysis of the complete plantar pressure pattern could offer additional valuable insights. Future work should now focus on investigating the validity and responsiveness of the proposed outcome measures.

## AUTHOR CONTRIBUTIONS

**Bente E. Bloks**: Conceptualization; data curation; formal analysis; methodology; visualization; writing – original draft preparation. **Lise M. Wilders**: Conceptualization; data curation; investigation; writing – review & editing. **Jan Willem K. Louwerens**: Conceptualization; writing – review & editing. **Alexander C. Geurts**: Conceptualization; writing – review & editing. **Jorik Nonnekes**: Conceptualization; data curation; methodology; writing – review & editing. **Noël L. W. Keijsers**: Conceptualization; data curation; methodology; writing – review & editing.

## CONFLICT OF INTEREST STATEMENT

None.

## ETHICS STATEMENT

Ethical approval was not required for this retrospective study. The study was conducted in compliance with the pertinent local ethical guidelines.

## Supporting information

Supporting Information S1

## Data Availability

Data sharing is not applicable to this article as participants did not give permission for this.
