# Supplementary material for: Plantar pressure in relation to hindfoot varus in people with unilateral upper motor neuron syndrome
Source: J Foot Ankle Res. 2024 Sep 2;17(3):e12041. doi: 10.1002/jfa2.12041 (PMC11369009; doi:10.1002/jfa2.12041)
Supplement: Supplementary file 1 — Supporting Information S1 [file JFA2-17-e12041-s001.pdf]

Supplementary material: statistical parametric mapping results

Figures S1 and S2 show the statistical parametric mapping post hoc test results performed on the center of pressure trajectories. In the medio-lateral direction, the center of pressure trajectory of the "no hindfoot varus" subgroup was significantly more medial between 83% and 92% of the stance phase compared to healthy controls ($p < 0.02$). For the "dynamic hindfoot varus" subgroup, the center of pressure trajectory was significantly more lateral compared to healthy controls in the first 26% of the stance phase and from 95% till the end of the stance phase ($p < 0.04$). For the "persistent hindfoot varus" subgroup, the center of pressure position was significantly more lateral during the first 82% of the stance phase compared to healthy controls ($p < 0.001$). Furthermore, the center of pressure trajectory was significantly more lateral for the "persistent hindfoot varus" subgroup compared to the "no hindfoot varus" subgroup between 16% and 79% of the stance phase ($p < 0.001$).

In the anterior-posterior direction, the center of pressure trajectories of all UMNS subgroups were more anterior during the first part of the stance phase compared to healthy controls ("no hindfoot varus": between 1-30% of the stance phase, "dynamic hindfoot varus": between 1-36% of the stance phase, "persistent hindfoot varus": between 1-30% of the stance phase, $p < 0.001$). Furthermore, later in the stance phase, the center of pressure trajectories of all UMNS subgroups were more posterior compared to healthy controls ("no hindfoot varus": between 44-77% of the stance phase, "dynamic hindfoot varus": between 53-80% of the stance phase, "persistent hindfoot varus": between 53-82% of the stance phase, $p < 0.001$). For the "no hindfoot varus" subgroup, the center of pressure trajectory was more anterior between 81% and 95% of the stance phase compared to healthy controls ($p < 0.003$).

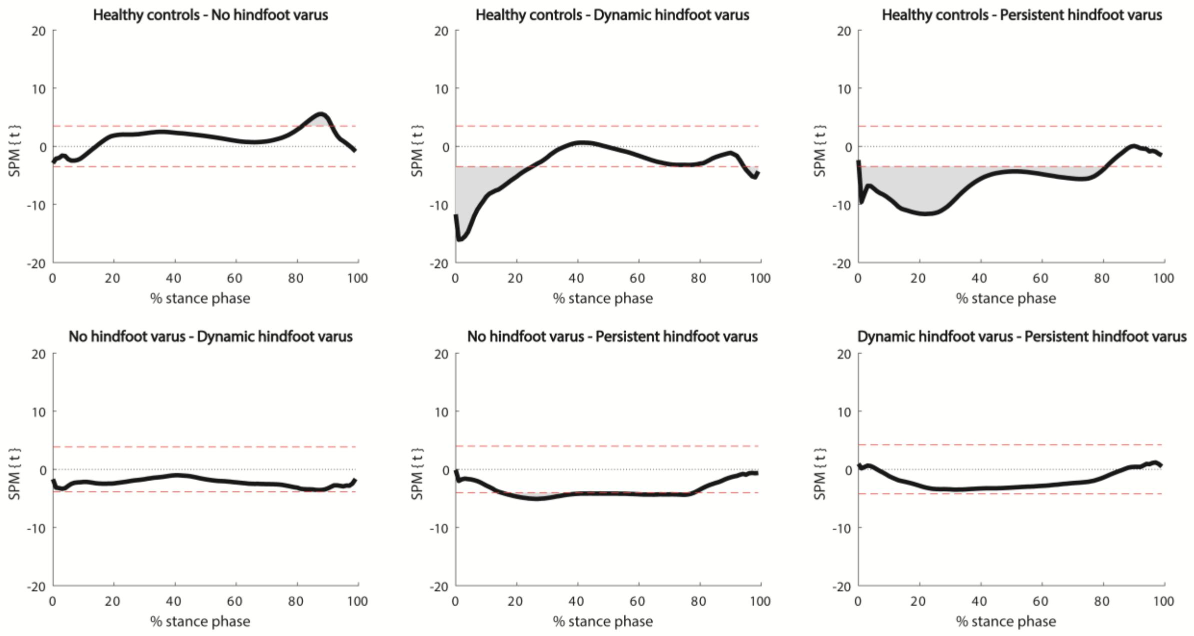

Figure S1. *Statistical parametric mapping post hoc test results on the center of pressure trajectory in the medio-lateral direction. Each panel presents the test statistics for a comparison between (sub)groups. In case the black line exceeds the critical t threshold (shown in red), the difference in center of pressure trajectory is statistically significant.*

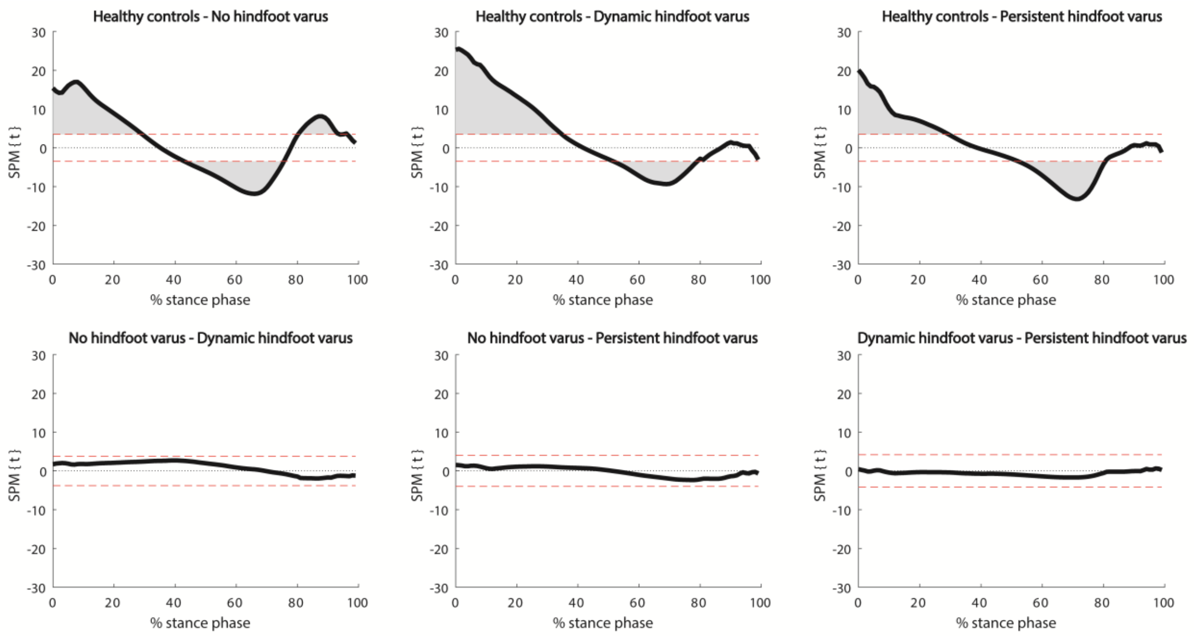

Figure S2. *Statistical parametric mapping post hoc test results on the center of pressure trajectory in the anterior-posterior direction. Each panel presents the test statistics for a comparison between*

(sub)groups. In case the black line exceeds the critical t threshold (shown in red), the difference in center of pressure trajectory is statistically significant.
